# Evaluation of XR device’s real-world tracking accuracy and depth perception from an industrial point of view

**DOI:** 10.1007/s10055-025-01192-3

**Published:** 2025-07-22

**Authors:** Shubham Singh, Yash Sharma, Amer Liaqat, Roy S. Kalawsky

**Affiliations:** 1 AVRRC, Loughborough University, Epinal Way, Leicestershire, LE11 3TU England, UK; 2 RNT, Airbus UK, Broughton, Chester, CH4 England, UK

**Keywords:** Virtual reality, Tracking accuracy, Evaluation methods, XR head mounted display, Inside-out tracking, Motion capture

## Abstract

The advancements in the field of XR devices and systems are interesting from an industrial point of view, as they present new opportunities for improving productivity and operations through—smart tooling, digitally enhanced assembly and maintenance, inspection, remote collaborations, etc. Typically, the XR headsets claim to provide a full 6-DoF tracking, while this may be good enough for consumer or entertainment applications; for an industrial application, we need to determine the exact errors and tolerances of the tracking for practical applications. In this paper, we present our methods and critical measurements from evaluating HTC Vive XR Elite and Magic Leap 2 for full 6-DoF tracking, depth perception accuracy, and drift accumulation over time. Through these tests, we measured a significant difference between individual XR devices’ tracking accuracy, depth perception, and drifts, which could range from moderate to severe impact for the on-job deployment of these devices. By systematically analyzing error margins and tracking fidelity, this study aims to provide new valuable insights into the strengths and limitations of tracking capabilities of these XR devices, and the methodology which can be adopted to evaluate others. Further, this study could also help design AR symbology and user experience for an industrial application.

## Introduction

Extended Reality (XR) technologies, comprising of virtual reality (VR), Augmented Reality (AR), and Mixed Reality (MR), are increasingly being adopted across a range of industries, from entertainment and gaming to industrial and enterprise applications (Raji et al. 2024; Jalo et al. 2022; Doolani et al. 2020; Torro et al. 2021). A cornerstone of XR functionality is the precise 6 Degrees of Freedom (6-DoF) inside-out tracking system, which allows a device to accurately track both its position (X, Y, Z coordinates) and orientation (pitch, yaw, roll) in real-time 3D space. This capability is essential to ensure that virtual objects and environments are rendered seamlessly in relation to the physical environment of the user.

**Fig. 1:**
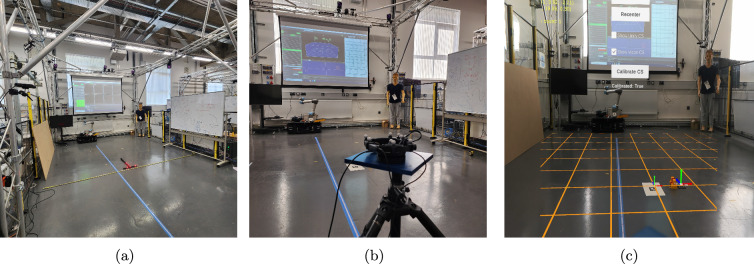
Vicon based XR tracking setup used for the evaluation of XR devices 6-DoF tracking accuracies. (**a**) Physical tracking space of 6 m x 4 m with the origin defined using Vicon calibration wand; (**b**) XR HMD (ML2) fitted with Motion capture markers for external tracking; (**c**) Virtual coordinate space aligned with physical (or Vicon) coordinate space, with the help of an AprilTag image target.

While casual entertainment and immersive XR apps often prioritize user engagement and realism, industrial applications require far stricter requirements on the precision and stability of 6-DoF tracking. In settings such as manufacturing, architecture, engineering, and healthcare, even small inaccuracies in pose tracking can lead to costly errors, safety concerns, and operational inefficiencies. For instance, an AR-guided assembly in a manufacturing environment requires precise alignment of virtual instructions or overlays with real-world components. Any drift or inaccuracy in anchor positioning could result in misalignment, leading to potential defects in production or machine calibration.

The accuracy and consistency of pose tracking become even more critical when industrial applications depend on long-duration interactions. Anchor drift—where virtual anchor points shift away from their real-world counterparts over time can degrade the performance of these systems. In contrast to casual XR experiences, where minor drift may go unnoticed or be tolerable, industrial use cases demand sustained precision over extended periods, as inaccuracies can directly impact operational performance and decision-making.

Most commercially available XR devices use a combination of sensors like—cameras for visual tracking; accelerometers and gyroscopes for motion sensing; and occasionally, depth sensors or LiDAR for Spatial mapping. Despite the sophisticated sensor fusion and an array of sensors, inaccuracies and drifts can always arise (Grimm et al. 2022; Hu et al. 2024). Environmental factors like poor lighting and dynamic scene changes can disrupt feature detection and tracking. Intrinsic camera flaws, such as lens distortion and sensor noise, introduce errors. Real-time tracking algorithms, essential for computational efficiency, may suffer from approximations and delays that accumulate over time. Moreover, external factors like occlusion and reflective surfaces create ambiguous visual cues, further challenging accurate 6-DoF tracking. Collectively, these issues contribute to the observed inaccuracies and drifts in XR devices.

While in the past there have been several studies conducted on measuring the tracking accuracy, we observed that most studies were orchestrated in some restricted manner, like—using a robotic arm (Jost et al. 2019; Sitole et al. 2020; Eger Passos and Jung 2020) or smaller scale tracking space (Verdelet et al. 2019; Maciejewski et al. 2018) on either HTC Vive Tracker (Outside-In tracking) or Meta Quest (Holzwarth et al. 2021). This paper presents our test results of a user walking freely in an open factory shop floor like environment within a tracking space of 6 m x 4 m, on Magic Leap 2 and Vive XR Elite devices. It is important to conduct these tests in an industrial environment setup to reproduce the same challenges that will be faced during actual deployments, like—large open spaces, dynamic environments with unrelated activities, and naturalistic user movements. Prior studies suggest it’s critical to evaluate AR systems in an actual manufacturing environment for any real-world industrial application (Zhang et al. 2024).

The accurate pose registration and perception of virtual objects in VR/AR remains a critical challenge. Previous studies show that AR systems offer better depth perception compared to VR (Ping et al. 2019; Westermeier et al. 2024), with a varying perception error at different levels of depths (Swan et al. 2006; Livingston et al. 2009). Further, the XR rendering system adopts multiple rendering and post-processing effects like– stereoscopic parallax, geometry or lens distortion correction, etc. which creates the illusion of depth for an immersive experience. Accurate depth perception relies on the proper alignment and rendering of slightly offset images to each eye, mimicking how the human visual system naturally processes depth information. However, when artifacts are introduced, they can distort this depth information, leading to misinterpretation of distances and object placements. This can cause objects to appear closer or farther than intended, creating unnatural or inconsistent depth cues. As the perceived depth increases, these discrepancies become more pronounced, since larger disparities between the images for each eye are required to convey distant objects. Inaccurate depth cues at larger distances may lead to a "flattened" or exaggerated sense of depth, making distant objects difficult to gauge precisely. This paper provides a new methodology for evaluating depth perception at varying depths and test results from optical and video pass-through devices.

This paper examines the pose-tracking capabilities of mainstream XR devices including both the optical and video pass-through display, with a focus on their practicality for industrial applications. This paper specifically evaluates how well these devices sustain accurate 6-DoF tracking, depth perception, and manage drift over time. These findings provide essential insights into the suitability of these commercial XR devices for critical real-world use cases and outline a methodology that can be adopted to assess other OpenXR^1^ compliant XR devices.

## Methodology

The testing apparatus included a Vicon motion capture system (v3.7) comprising 8 rail-mounted Vero cameras covering an area of 6 m x 4 m over an open floor space. Two XR devices were selected for this evaluation—Magic Leap 2 (here onward referred to as ML2) OS version 1.9.0, and HTC Vive XR Elite (here onward referred to as Vive XR) version 7.3 (System v1.0.999.702), having an optical and video pass-through display respectively. More details about their display and tracking systems are provided in Table 1. The XR application was developed in Unity v2022.3LTS, using OpenXR SDK (Software Development Kit) and installed on both devices.

**Table 1:** XR device specification for tracking and HMD

	Vive XR elite	Magic leap 2
Device type	Standalone VR	Standalone AR
Optics	Binocular pancake lenses	Binocular waveguides
Display	2 X liquid crystal display (LCD)	2 X liquid crystal on silicon (LCoS)
Resolution	1920x1920 per-eye	1440x1760 per-eye
Refresh rate	90Hz	120Hz
Tracking type	6-DoF inside-out via 4 integrated camera	6-DoF inside-out
Base stations	✗	✗
Eye tracking	✗	✓
Hand tracking	✓	✓

The Vicon tracking system was used to provide the ground truth value of the XR device position and rotation, which is accurate to a sub-millimetre level of precision (Merriaux et al. 2017). Vicon motion capture system utilize high-speed cameras and reflective markers to track and record precise 3D movement data. The cameras detect infrared light reflected from markers placed on subjects, allowing the system to calculate their pose in real-time. Thus offering high accuracy and reliability, making them essential for quantitative motion analysis in scientific studies. Both the XR head-mounted devices (HMDs) were affixed with mocap markers (See Fig. 1b) and pose data for the tracked object was polled via Vicon Datastream SDK over the local wireless network.

**Fig. 2:**
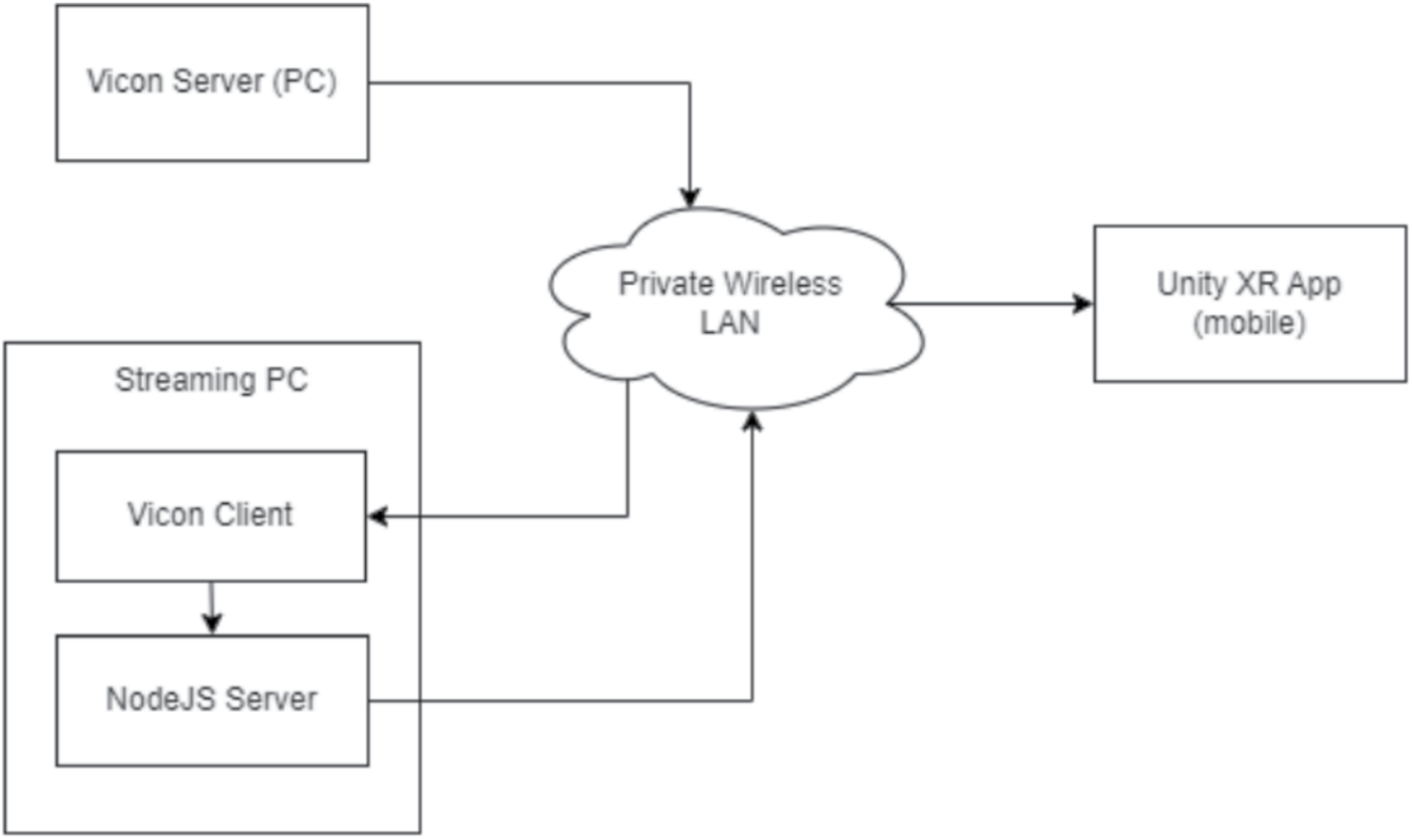
High-level communication architecture between Vicon and mobile XR application

Since the Vicon Datastream SDK is only available for standalone Windows, MacOS, or Linux platforms, the tracked information couldn’t be directly accessed over mobile XR devices. This necessitated the requirement of an intermediate bridge application that can forward the received data packets over the local network wirelessly. A high-level communication architecture diagram is provided below for reference (See Fig. 2).

For the XR application to be deployed on devices the Unity3D development environment was used, which is freely accessible and supports cross-platform development for a wide variety of XR devices. Additionally, to be able to use the same codebase across all major XR devices the OpenXR SDK and compliant devices were used. Which avoids the need for developing new applications with the native SDK for each new device. By adhering to OpenXR standards and specifications we can be fairly confident that the same project can be deployed to new untested devices out there, or those that may come in the near future.

Normally, in Unity depending upon the XR-device SDK the virtual coordinate space is defined based on aspects like– application starting point or external room setup process beforehand. Whereas in Vicon the coordinate space is explicitly defined with respect to the physical space with the help of a calibration wand (see Fig. 1a). Thus for comparing the tracking data from two independent systems (Vicon and XR), we define a new coordinate space which can be reliably tracked/referenced by each of the tracking systems. Then we estimate the respective transformations between the different coordinate spaces to align and compare values. This calibration step is always performed before capturing any data.

To define a new common coordinate space we used an additional fiducial marker or object tracking available from the device SDK. In the case of ML2, we used Image Tracking and for Vive XR we used the Vive Ultimate Trackers, which are used to define a new virtual coordinate space that coincides with the Vicon Origin and is aligned in the same direction. The idea is to align these independently tracked marker/object with actual Vicon origin, which can be reliably used to transform any vector from a virtual XR space to Vicon-tracked real-world space and vice versa.

### Design

All the tracking tests were conducted in an indoor open floor space with a tracked area of 6 m x 4 m. The room had concrete flooring with a dark greyish colour and specular finish. The indoor space was primarily lit by LED ceiling lights with additional ambient light coming through blinded large windows. The lab space was chosen to closely simulate the real factory shop-floor environment as much as possible.

Before starting any test application, the Vicon setup was freshly calibrated with an average world error of the system to be approx. 0.2971 mm. The Vicon tracking system is excellent for any real-time application (Merriaux et al. 2017), with the system’s tracking latency of 10 ms (or 100 Hz frame rate). While we expect the actual frame rate to vary slightly over a wireless network through the streaming SDK, in our experience, perceptually there was no noticeable issue/delay in real-time visualization of the tracked data in XR.

A total of three experimental tests were undertaken for the evaluation of the suitability of these XR devices:

- 6-DoF Pose tracking. A quantitative test comparing the pose updates reported by XR SDK and Vicon streaming SDK, in a commonly referenced coordinate space.
- Depth perception. A qualitative assessment of virtual depth perception accuracy at multiple levels of depth in XR.
- Virtual anchor drift. A quantitative test to measure how much a virtually placed anchor in 3D space drifts over time.

#### 6-DoF pose tracking

Firstly, the virtual Vicon coordinate space (inside Unity) is aligned with the desired physical space with the help of a tracked marker/object. In the case of ML2, we used the AprilTag image target, which is placed such that it coincides and aligns with Vicon’s defined Origin. For Vive XR Elite, since there’s no option for doing image tracking in the default SDK, we relied on Vive Ultimate Trackers, which can be tracked independently in a 3D space. Once we were fairly confident that the tracked image/object is placed at the origin, we performed the calibration step to calculate all the transformation matrices between different spaces—Vicon-defined physical space, Unity-based virtual XR space, and newly defined tracker-based virtual coordinate space.

Note that despite the best effort of positioning and aligning the XR tracked target with Vicon’s Origin, there is always a possibility of fixed offset between the XR HMD pose and Vicon HMD pose due to– different pivot points for tracking, and human error in positioning the tracking target over the origin. Thus, alongside the Transformation matrix, another offset matrix was calculated for fixed position and rotation offset, which is always applied to Vicon received pose data after the transformation. Figure 1 (c) which shows the orange Vicon coordinate space aligned with physical Vicon space resulting in a virtual axis anchor being placed over the Vicon tracked object plate (placed on the right of Lego bricks).

Thus for any vector $v_{xr}$ in virtual coordinate space can be converted to physical/Vicon coordinate space vector $v_{re}$ using 4x4 transformation matrix $M: \Re ^4 \xrightarrow {} \Re ^4$, such that:

1 $$\begin{aligned} v_{re} = M \cdot v_{xr} \end{aligned}$$

where $M \in SE(3)$ (i.e. Special Euclidean Group) representing rigid body motion of independently tracked object/target with 6 degrees of freedom. Next, for calculating the HMD offset $M_{off} \in SE(3)$ between XR and Vicon:

2 $$\begin{aligned} M_{off} = V_{world\_to\_local} \cdot U_{local\_to\_world} \end{aligned}$$

where $V_{world\_to\_local}$ is the Vicon’s HMD transform matrix for converting any virtual world space to HMD object’s local space and $U_{local\_to\_world}$ is the XR HMD transform matrix in virtual world space. Thus essentially giving us a 4x4 matrix representation of XR HMD offset in Vicon HMD’s local space.

Additionally, there is always a chance of network data packet delay or corruption; hence, while recording the pose value of transformed XR-HMD and Vicon-HMD, we use an averaged pose value over a fixed number of consecutive frames (10 frames) over time.

**Fig. 3:**
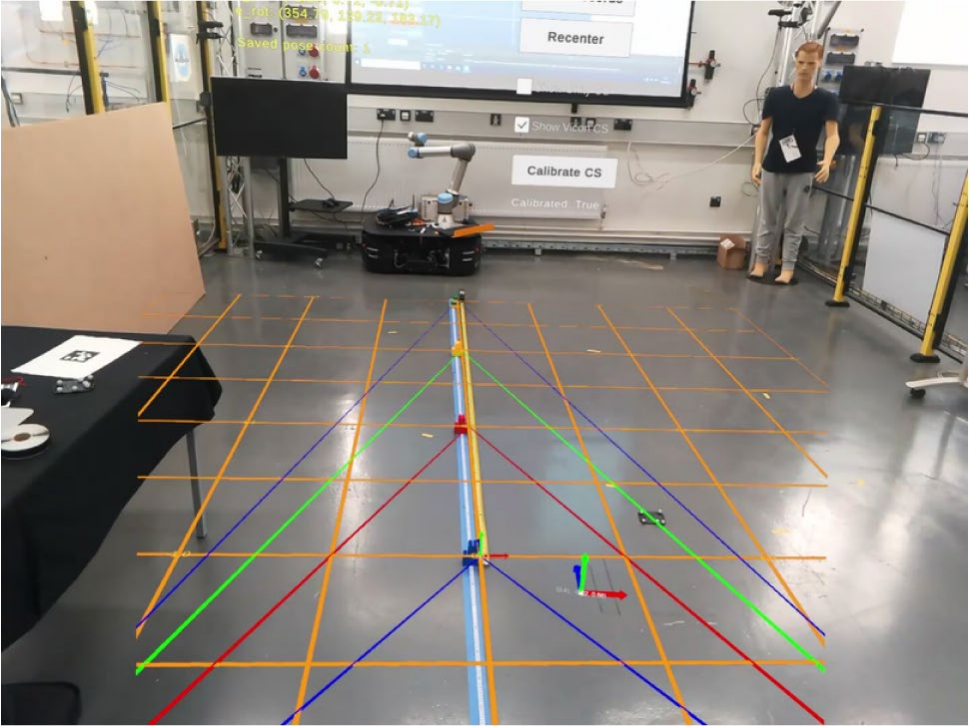
Depth perception disparity measurement between virtual and real-world using measuring tape on ML2 device. With vantage point being at (0,-2), and subsequent converging points starting from (0,0), to (0,1), (0,2), and (0,3) along the z-axis

#### Depth perception

For measuring depth perception, we designed a perceptual matching arrangement with a few converging lines at different depths from a fixed vantage point on the XZ grid plane (See Fig. 3). The figure shows XZ grid plane represented via orange lines, and converging lines with different red, green, and blue, colours. The point where the two lines meet we called this point as a converging point, which is designed to be at a 1-meter distance apart between the consecutive points. The hypothesis is that, at increasing depth value the virtual distance would either be lesser or greater than the actual distance. This information is important from an operational perspective, as this would determine the tolerance for information overlays at different distances from the user. Note that due to stereo rendering textures and the compositing step of combining virtual texture with camera image, the captured snapshot looks different from what the user perceives during the experience. This disparity between the captured image and what the user sees during the experiment is even more prominent on the video pass-through of Vive XR.

During the test, we adopted two strategies for placing the Lego blocks at the point of convergence at different depths. Firstly, a near placement method, where the user goes close to the point of convergence and places the blocks themselves. Secondly, a far placement method, where the user stands at the vantage point and guides the non-immersed person to position the block at the perceived location of the point of convergence. In the second placement method, the user is allowed a translational movement of up/down but not left/right or forward/backwards, to maintain the z-depth. The idea behind the two strategies is that with the near placement, we alleviate any depth perception so it should be significantly closer to true depth in comparison to the one placed during far placement. Thus, providing a new reference measurement for comparison.

#### Virtual anchor drift

All spatial tracking systems suffer from drift, which is caused due to the accumulation of small errors over time (Scargill et al. 2021). For XR this results in the virtual anchor moving away from where it was originally placed. However, with loop-closure and relocalization techniques, the system can correct itself for extended operation duration. To measure this, we simply place multiple virtual anchors overlayed on top of the real-world measuring tape and record their position on the measurement tape during the placement step. Then freely moving about the tracked space for roughly 2-3 min and re-record the virtual anchor position on the measuring tape.

## Results

Following the described systematic methodology and development, below are the results for each of the experiments on ML2 and Vive XR devices, using a Vicon tracking system for ground truth values.

### 6-DoF pose tracking

Table 3, presents the scatter plot of the HMD positions (X, Y, and Z) and rotation difference (pitch, yaw, and roll) between XR and Vicon, for two tests 1 and 2. In Test 1, the XR device was moved across the XZ-plane in a zig-zag pattern from +X to −X across the Z axis, whereas in Test 2 it was moved across the grid in a long diagonal pattern with a larger gait. In the first row of the position error plot, the size of the red dotted circle indicates the distance error between XR-HMD and Vicon-HMD, with the respective XR position indicated with a ’$+$’ symbol in a top view. The distance error is calculated as:

3 $$\begin{aligned} {d = \sqrt{(x_2-x_1)^{2} + (y_2-y_1)^{2} + (z_2-z_1)^{2}} } \end{aligned}$$

**Table 2:** Positional error statistics for ML2 and Vive XR

		Min error (mm)	Max error (mm)	Mean error (mm)	Standard deviation $\sigma$(mm)
ML2	Test 1	9.117	260.149	66.380	41.368
	Test 2	15.850	142.103	69.996	34.455
Vive	Test 1	36.255	251.295	123.778	55.748
	Test 2	7.848	249.739	125.243	66.676

**Table 3:**
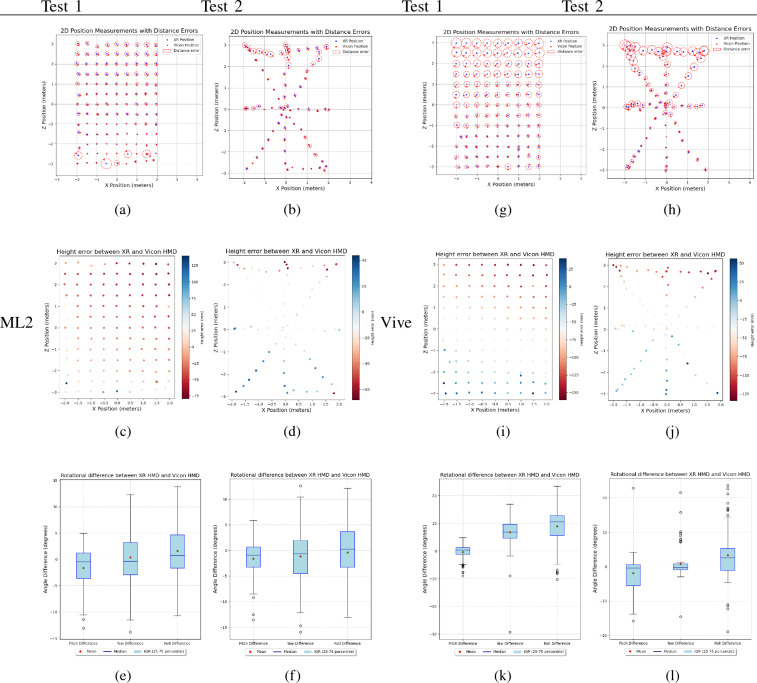
6-DoF pose error plot for test 1 and test 2 for ML2 and Vive XR. The first row (graphs a, b, g and h) presents the XZ position error scatter plot on a 4 m x 6 m XZ plan.The second row (graphs c, d, i, and j) presents the height error (along the Y-axis) scatter plot. The third row (graphs e, f, k, and l) presents the box plot for angular difference (pitch, yaw, and roll) between XR and Vicon HMD

Here it can be noted that in the case of Vive XR, the size of the circle increases as we go further away from the origin. In the case of ML2, it’s much smaller with occasionally large error values, possibly due to the XR device updating the local map or re-localizing after some time. Table 2 presents the positional statistics from the two sets of tests, indicating that ML2 offers much better positional accuracy with $68.188\pm 37.911$ mm average distance error, as compared to Vive’s $124.510\pm 61.212$ mm.

Next, from the height error plot (figure c, d, i, and j in Table 3), we observe that in the case of ML2, the height error dots are mostly either less (test 1) or more (test 2) than the true height of the ground plane. Whereas in the case of Vive XR (graphs i and j), we observe that in both test cases, roughly half of the dots represent a negative errors (red colour) while the remaining half a positive errors (blue colour).

From the last row of Table 3, we observe that ML2 tracking has a rotational error of $1.125\pm 4.768$ degrees on average, while Vive XR has an average rotational error of $3.695\pm 5.311$ degrees.

### Depth perception

**Table 4:** Depth perception test with near and far placement on ML2 and Vive XR Elite

	Conv. point (virtual distance from vantage pt in cm)	Near placement		Far placement	
		Position (cm)	Error (cm)	Position (cm)	Error (cm)
ML2	0(200)	3.5	0	12.9	0
	1(300)	103.6	0.1	110.9	− 2.0
	2(400)	203.2	− 0.2	204.4	− 8.5
	3(500)	302.8	− 0.7	305.6	− 7.3
Vive	0(200)	5.8	0	20	0
	1(300)	103.7	− 2.1	106.5	− 13.5
	2(400)	201.7	− 4.1	205.3	− 14.7
	3(500)	301.5	− 4.3	300.8	− 19.2

Table 4, presents the results of the depth perception test measurement, with near and far placement strategy. Note that the error calculation for Near and Far placement is calculated based on the relative expected distance from the starting virtual point of convergence 0, with each subsequent point having a consecutive gap of 1 m.

### Virtual anchor drift

**Table 5:** Anchor drift test along X and Z axes of XZ-ground plane for ML2 and Vive XR.

		Placement position (cm)	Observation position (cm)	Drift amount (cm)
ML2	Anchor_x_0	9.5	9.6	0.1
	Anchor_x_1	59.8	59.9	0.1
	Anchor_x_2	260.2	260.2	0.0
	Anchor_x_3	360.3	360.2	− 0.1
	Anchor_z_0	59.4	59.4	0.0
	Anchor_z_1	159.8	159.8	0.0
	Anchor_z_2	359.9	359.9	0.0
	Anchor_z_3	460.2	460.4	0.2
Vive	Anchor_x_0	4.3	3.2	1.1
	Anchor_x_1	99.2	104.2	5.0
	Anchor_x_2	295.1	298.9	3.8
	Anchor_x_3	395.4	402.1	6.7
	Anchor_z_0	14.2	14.4	0.2
	Anchor_z_1	112.1	112.5	0.4
	Anchor_z_2	310.9	312.5	1.6
	Anchor_z_3	407.2	412.5	5.3

The position values indicate the virtual anchor’s placement over a physical measuring tape line

Table 5, presents the anchor drift test results for ML2 and Vive XR. Between the two devices, we observe that ML2 has better anchor locking with an average drift value of 0.6 mm when compared to Vive XR, which has an average drift value of 30.1 mm.

## Analysis

### 6-DoF pose tracking

Following the pose tracking results from Tables 3 and 2, it is noted that ML2 did better than Vive XR in all aspects of 6-DoF pose tracking—positional error, height, and rotational error. In terms of average positional error, ML2 had a lower value of 68.188 mm with a standard deviation of 37.911 mm compared to Vive which had 124.510 mm with a standard deviation of 61.212 mm. Similarly, for rotational errors ML2 did significantly better, having an error of $1.125^{\circ }$ with a standard deviation of $4.768^{\circ }$ as compared to Vive having $3.695^{\circ }$ and a standard deviation of $5.311^{\circ }$. Further, from the Boxplot graph, we observe that the Vive rotational value had a relatively larger number of outliers as compared to ML2, indicating fluctuations in tracking due to either losing track or drifts.

With respect to the height error plot, it is noted that the ML2 graph has mostly either blue (positive error) or red (negative error) dots, indicating consistent height difference between the virtual ground plane in XR and the physical ground plane. Comparing this to Vive XR we found that the virtual ground plane in XR was inclined at an angle with respect to the physical ground plane thus resulting in roughly half of the height error dots being of blue colour while the remaining dots being of red colour.

### Depth perception

For the depth perception test, we expected the near-placement results to be significantly better than the far-placement approach, and in far-placement, the users to either under or over estimate the depth cues depending on the pass-through type. From the results, we notice that for both the ML2 and Vive XR, the near placement of the blocks closely matches the expected gap of 1 m between each consecutive point and is significantly less than far placement error values. Subsequently, the error values in near placement can be attributed to factors like—device tracking, quality of pass-through, and human perception.

Further, for the far placement approach, it is noted that the depth perception error increased with increasing levels of depth, and ML2 provides significantly better depth perception with an error value of $\sim$7.3 cm compared to Vive XR error of $\sim$19.2 cm, at the depth of 5 m in front of the user (or vantage point). These results are in line with our expectation that optical pass-through devices offer better depth perception as compared to video pass-through devices.

### Virtual anchor drift

For the anchor drift test, it was interesting to note that in the case of ML2, despite the test anchor being placed across the full length and breadth of the XZ-plane, while revisiting the previously placed anchor-point location, there was little to no drift observable. We think this is due to good relocalization and tracking of the ML2 SDK. However, Vive XR continued to fall short in comparison, having a maximum drift amount of 6.7 cm.

## Discussion

The findings presented in this study offer crucial insight into the real-world implications of XR tracking performance, particularly in XR-based assistive industrial and manufacturing workflows where precise spatial alignment is critical. The observed error margins in the tracking systems can have direct consequences on the usability and reliability of XR applications in these settings. For instance, in manufacturing tasks where real-world spatially referenced augmented instructions such as markers or targets are overlaid on physical components, even little errors in anchor placement could lead to misalignment, increasing the likelihood of assembly mistakes and reducing structural efficiencies.

It is important to note that our results for the error margin values are significantly greater than the findings of Jost et al. (2019) and closer to Holzwarth et al. (2021), particularly when considering just Vive tracking. Although this may be due to a change in device type, SDK version, or tracking system (Inside-Out vs. Outside-In), this also highlights the importance of conducting these evaluations and tests in a real factory environment with naturalistic user movements.

Similarly, it is interesting to note that the ML2 used in our study offered a better depth perception error of 7.3cm for an optical pass-through display at a depth distance of 5 m compared to  20 cm on NED AR HMD used by J Ping et al. (2019). Although these results are better for ML2, on Vive (video pass-through device), it is similar to their results, highlighting the importance of conducting these tests individually on respective devices. These error margins are still significant and should be considered in high-stakes jobs. Any misjudgments of depth perception or anchor inaccuracies could result in operational errors, incorrect diagnostics or guidance, or safety hazards.

In industrial environments where XR is deployed for ongoing operations to mitigate tracking issues, developers might consider incorporating methods like—frequent recalibration; sensor fusion techniques; or adding external tracking sources that work in tandem with onboard inside-out tracking for higher precision. In addition, the depth perception error is inherent to the XR system’s runtime and display optics. As we observe, depth perception degrades increasingly with distance. So, developers might adopt preventative steps like not showing visual cues and overlays beyond the tolerable ranges, or adopting symbology that indicates anchor placement uncertainties, etc. Moreover, XR augmented symbology presented at short ranges (where the distance to the object of interest is less than 1 m) may cause user depth parallax issues if the optical system is designed for a different accommodation distance. While some of this can be accounted for in the stereo disparity at the software level, there is a finite limit. The age profile of the user is also an important factor here. As we get older our ability to accommodate a wide range becomes more limited, making it difficult to resolve the depth cues.

From an end-user perspective, understanding these limitations is essential for designing effective XR applications to be used in industrial settings for operational or training use cases. Workers who rely on XR interfaces should be educated on the potential for tracking inconsistencies and trained in best practices for recalibrating or verifying spatial accuracy. Furthermore, deploying XR systems with built-in feedback mechanisms, such as visual symbology and indicators that highlight potential tracking errors, could enhance user awareness and decision-making in industrial contexts.

Thus, while XR tracking technologies offer transformative potential across various industries, their deployment requires careful consideration of error margins and system limitations. By integrating real-time corrections, leveraging multi-sensor approaches, and educating users on best practices, developers and industries can maximize the effectiveness of XR applications in high-stakes, precision-dependent environments.

## Conclusion

In this study, we presented the methodology and results to evaluate the suitability of mainstream XR devices—ML2 and Vive XR for industrial application, via the state-of-the-art Vicon motion capture system. Following the analysis from three experiments, it is noted that ML2 offers better tracking capabilities, depth perception, and a lower anchor drift over time compared to Vive XR. In the 6-DoF pose tracking test, ML2 offered twice (average position error of $\sim$6.9 cm) as good translational tracking as compared to Vive XR (having average position error of $\sim$12.5 cm), with rotational error of $1.125^{\circ }$ versus average error of $4.768^{\circ }$ in Vive XR. In the depth perception test at a maximum view distance of 5 m, ML2 had a better depth accuracy with error of 7.3 cm as compared to error of 19.2 cm on Vive XR. Lastly, for the drift test on ML2, a max drift of 0.2 cm was recorded when compared to 6.7 cm on the Vive XR.

Whilst, ML2 did well analytically on all tests, we generally had more usability issues with ML2 during the experiments. The device was not particularly comfortable to be worn over spectacles, with no feasible option for prescription lenses, as compared to Vive’s adjustable diopter dial for each eye. Another major operational problem was that the ML2 device rebooted without any prompt or notification when it lost track of the space. In our view, this is a serious limitation that results in the loss of any active progress or operation in the middle of a session. Furthermore, it was easier to lose track on the ML2 than on the Vive XR in our lab environment, and the Vive XR recovered the tracking more easily.

In conclusion, this study highlights the limitations of 6-DoF tracking claimed by commercial XR devices, while highlighting the importance of conducting these tests on a factory shop floor environment for industrial applications. Knowing these pose errors and depth perception limits, we can adopt counter measures, for example—using external tracking to correct or locate the XR devices in a large area; or modifying AR Symbology and user experience to present meaningful and spatially accurate information (i.e. not showing infographics or indicators beyond the tolerable margin of error).

## Footnotes

1 OpenXR is a royalty-free, open standard maintained by Khronos group, that provides a common set of APIs for developing XR applications that run across a wide range of AR and VR devices Khronos (2025).
